# Supplementary material for: Artificial Neurons on Flexible Substrates: A Fully Printed Approach for Neuromorphic Sensing
Source: Sensors (Basel). 2022 May 25;22(11):4000. doi: 10.3390/s22114000 (PMC9182789; doi:10.3390/s22114000)
Supplement: Supplementary file 1 [file sensors-22-04000-s001.zip › sensors-1682364-supplementary.pdf]

Supplementary Material - Artificial Neurons on Flexible Substrates: A Fully-printed Approach for Neuromorphic Sensing

Contact Angle

In order to print structures in the desired dimensions, it is important to study the surface effect of the substrate on the printed ink. We observe the contact angle made by the printed drops while printing graphene ink as well as the precursor of In_2O_3. The contact angle is measured using a sessile goniometer and the contact angle is calculated on the drop to test the wettability. The contact angle is calculated by equating the interfacial and surface tensions as per the Young's equation 5:

$$\sigma_{sg} = \sigma_{sl} + \sigma_{lg} \cdot cos\theta \quad (5)$$

with the surface free energy of the substrate σ_{sg}, the interfacial tension between the ink and the substrate σ_{sl}, the surface tension σ_{lg}, and being the contact angle θ.

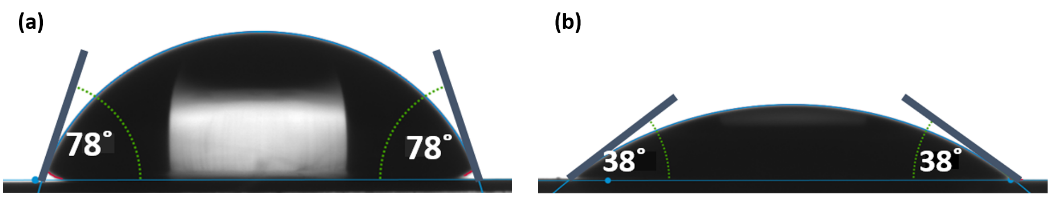

Figure S1. Contact angle of **(a)** graphene on polyimide, and **(b)** indium nitrate precursor on polyimide. Note that in each case the polyimide has been first subject to 400 °C heating followed by isopropanol-acetone cleaning step.

Surface Morphology

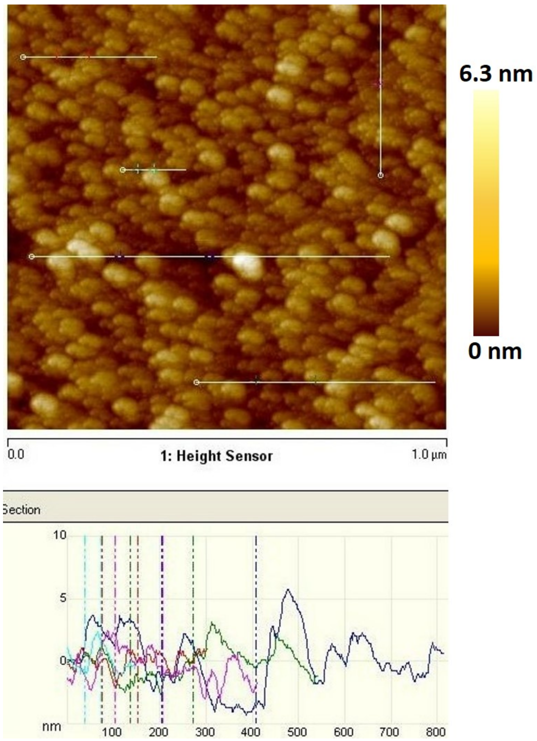

Figure S2. Atomic force micrograph scans performed on the In_2O_3 channel in various directions to check for surface morphology. A maximum roughness of 6 nm is observed without any cracks, with a standard deviation of about 2 nm.

Owing to the difference in the roughness of graphene and indium oxide as well as the difference in thermal expansion coefficients, there is a possibility for abrasion. This is avoided by slowly annealing both the layers together. SEM and AFM imaging of the interface shows no visible abrasion.

EDX Analysis after Bending

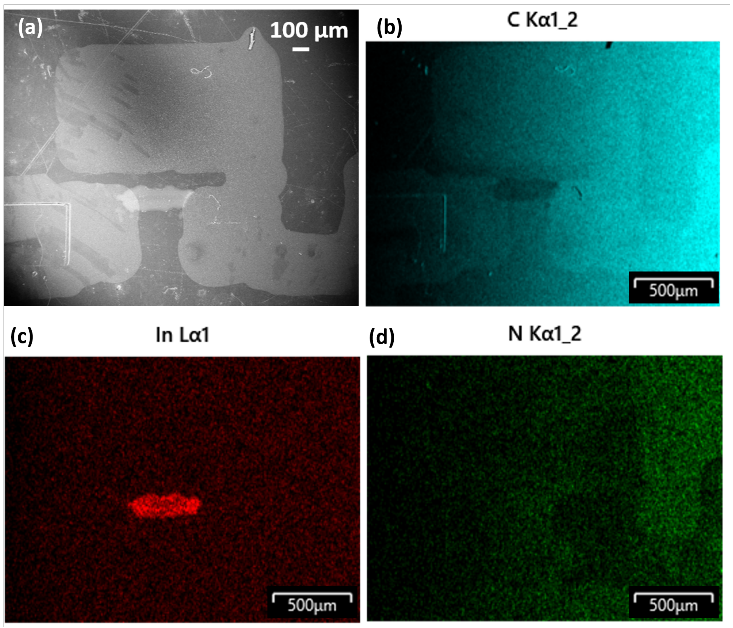

Figure S3. Electron dispersive X-ray mapping of the EGT based ANN bent at 4.5 mm. (a) SEM image with corresponding EDX measurements of carbon (b), indium present in the semiconductor (c), and nitrogen present in polyimide (d)

Ageing

Transfer characteristics of the electrolyte gated transistor were measured initially and after four weeks. The measurements were performed at 50% humidity and clearly show no degradation of performance. The threshold voltage only slightly increased, which is expected due to charge trapping over time.

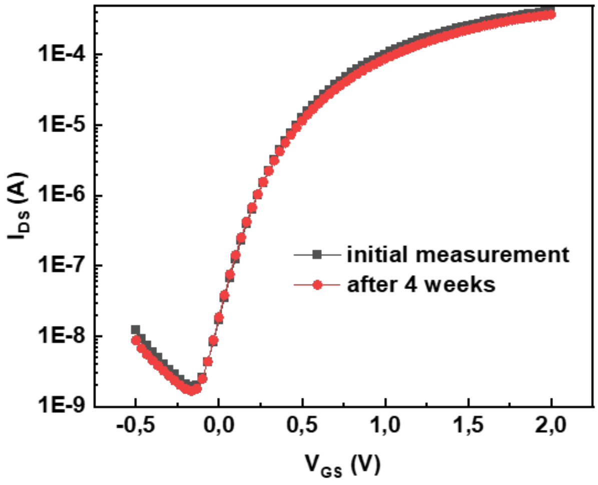

Figure S4. Ageing effect on the electrolyte gated transistor. Transfer characteristics initially and after four weeks match exactly with each other.

Power Consumption

To calculate the power consumption, the currents of this electric network are derived from the applied and measured voltages and the known resistances. The input voltages are applied to the pads V1 and V2, and the internal voltages are measured from Vx and Vout. The course of the input voltages is presented in Figure 7(a), the transient currents are computed. Finally, having both voltages and currents, the component-wise power consumption can be calculated and subsequently the total power consumption. As mentioned before, the most power is consumed by the MAC circuit, as the other part of the neuron with the diode and R_{out} have much higher resistances. The order of magnitude of the high power consumption of 37.7mW can be illustrated by the following back-to-envelope calculation: The maximum voltage across the MAC circuit is

$$V_{\max} = V_1 - V_2 = 2V - (-2V) = 4V$$

We have in total R_{max} = 500 Ω. The power consumption of the MAC circuit is thus

$$P_{max} = \frac{V_{max}^2}{R_{max}} = \frac{(4)^2}{500} = \frac{16V^2}{500\Omega} = 0.032W$$

Thus, the average power consumption is
Pavg = ½ Pmax = 0.032W = 32mW. In our fabricated neuron the printed components and voltages are imperfect, but the experimental outcome is close to this illustrative calculation.
